# Social Media News Use and COVID-19 Misinformation Engagement: Survey Study

**DOI:** 10.2196/38944

**Published:** 2022-09-20

**Authors:** Saifuddin Ahmed, Muhammad Ehab Rasul

**Affiliations:** 1 Wee Kim Wee School of Communication and Information Nanyang Technological University Singapore Singapore; 2 Department of Communication University of California Davis Davis, CA United States

**Keywords:** COVID-19, misinformation, personality, cognitive ability, social media, Singapore

## Abstract

**Background:**

Social media is widely used as a source of news and information regarding COVID-19. However, the abundance of misinformation on social media platforms has raised concerns regarding the spreading infodemic. Accordingly, many have questioned the utility and impact of social media news use on users’ engagement with (mis)information.

**Objective:**

This study offers a conceptual framework for how social media news use influences COVID-19 misinformation engagement. More specifically, we examined how news consumption on social media leads to COVID-19 misinformation sharing by inducing belief in such misinformation. We further explored if the effects of social media news use on COVID-19 misinformation engagement depend on individual differences in cognition and personality traits.

**Methods:**

We used data from an online survey panel administered by a survey agency (Qualtrics) in Singapore. The survey was conducted in March 2022, and 500 respondents answered the survey. All participants were older than 21 years and provided consent before taking part in the study. We used linear regression, mediation, and moderated mediation analyses to explore the proposed relationships between social media news use, cognitive ability, personality traits, and COVID-19 misinformation belief and sharing intentions.

**Results:**

The results suggested that those who frequently used social media for news consumption were more likely to believe COVID-19 misinformation and share it on social media. Further probing the mechanism suggested that social media news use translated into sharing intent via the perceived accuracy of misinformation. Simply put, social media news users shared COVID-19 misinformation because they believed it to be accurate. We also found that those with high levels of extraversion than those with low levels were more likely to perceive the misinformation to be accurate and share it. Those with high levels of neuroticism and openness than those with low levels were also likely to perceive the misinformation to be accurate. Finally, it was observed that personality traits did not significantly influence misinformation sharing at higher levels of cognitive ability, but low cognitive users largely drove misinformation sharing across personality traits.

**Conclusions:**

The reliance on social media platforms for news consumption during the COVID-19 pandemic has amplified, with dire consequences for misinformation sharing. This study shows that increased social media news consumption is associated with believing and sharing COVID-19 misinformation, with low cognitive users being the most vulnerable. We offer recommendations to newsmakers, social media moderators, and policymakers toward efforts in limiting COVID-19 misinformation propagation and safeguarding citizens.

## Introduction

The emergence of the novel coronavirus or SARS-CoV-2 wreaked havoc across the world. One of the consequences of the resulting pandemic was an unprecedented reliance on social media platforms, as public health agencies and governments turned to social media as a tool for news dissemination [1,2]. Therefore, social media platforms provided individuals with quick access to credible information while also allowing them to share their opinions and attitudes toward the pandemic [3,4]. Individuals also used social media to cope with additional stresses, stay-at-home orders, and remote work environments, while displaying what scholars consider to be signs of social media addiction [5]. The deluge of illegitimate, anecdotal, and emotional content created a perfect storm for the emergence of the COVID-19 infodemic involving the “undisciplined spread of information” [6,7], which swept through social media and was rife with rumors, disinformation, and conspiracy theories [8].

The effects of misinformation related to a destructive pandemic, such as COVID-19, are severe because false beliefs are difficult to correct [9], especially among those with low cognitive ability [10]. Moreover, although misinformation on social media is not a novel issue, it has become a key cause of concern owing to its pernicious impact on infectious disease management and public compliance with health protocols such as mask wearing [11]. For instance, researchers have found that misinformation on social media may lead to lower trust in public health authorities and the effectiveness of mitigation protocols [12]. In addition, studies have found that misinformation on social media fuels vaccine hesitancy through engagement with antivaccine beliefs [13,14]. The unabated spread of misinformation online, along with its severe impact on negative attitudes toward science and compliance toward public health protocols, has prompted scientific investigation into the sharing and engagement of misinformation related to COVID-19. However, the susceptibility to misinformation on social media varies from person to person. Scholars have argued that some individuals are more vulnerable to misinformation on social media than others [15]. While prior work has explored the relationship between general social media use and misinformation vulnerability, our concern is on the growing proportion of social media users who rely heavily on it for news updates. We argue that general social media use is distinct from social media news use. General social media use is a broad term that can encompass many activities (ie, liking posts, sharing posts, commenting, watching videos, etc). On the other hand, social media news use is a narrower operationalization of social media news consumption that can aid researchers in examining which specific aspects of social media use impact misinformation engagement. Indeed, a growing body of literature has found social media news use to be positively associated with the spread of conspiracy theories and misinformation [16]. Other studies have found social media news use to play a role in the sharing of deepfakes, a form of misinformation [17,18]. The post-COVID climate is likely to have a magnification effect on these relationships, as more and more people are increasingly turning to social media for news use. Therefore, we anticipate that social media news use offers a nuanced understanding of social media effects with potentially more serious consequences than previously understood.

The technological features of social media platforms play a role in the spread of misinformation on social media. For instance, algorithms that curate the social media feed seek to maximize engagement through prior behavior and clickbait [19]. Consequently, this may result in repeated exposure to misinformation and increase individual engagement with false information related to COVID-19. Existing research has established that repeated exposure to misinformation reinforces and increases trust in false beliefs [20]. Repeated exposure causes individuals to be more susceptible to misinformation through the illusory truth effect, which posits that repeated claims are seen as more truthful than nonrepeated claims [21]. Consequently, it may lead to poor discernment of truthful information and a lack of careful reasoning [22]. In addition to increased susceptibility toward misinformation, individuals may share false beliefs with others in their networks. As such, we hypothesize the following: *H1*, social media news use will be positively associated with (1) perceived accuracy and (2) sharing intentions of COVID-19 misinformation; and *H2*, the relationship between social media news use and sharing intention will be mediated by the perceived accuracy of COVID-19 misinformation.

Scholars have explored the individual-level differences in how people react to misinformation. For instance, prior research has found that political ideology, particularly conservativism, is a predictor and motivator of belief in misinformation [22-24] and of sharing COVID-19 misinformation–related conspiracy theories on social media [25]. Another study suggested that this may be because the heightened levels of anxiety among Republicans led them to trust and share misinformation related to COVID-19 on social media through partisan motivated reasoning and selective sharing [26]. However, the bipartisan American context may not apply to other contexts of social media use and misinformation sharing worldwide. Subsequently, we consider other intergroup differences related to reasoning and rationality that have been explored in the literature. For instance, a recent study found that people with higher levels of analytical thinking were less likely to believe and share COVID-19 misinformation on social media [27]. Likewise, researchers have pointed out that individuals with high cognitive ability are less vulnerable to misinformation on social media [28]. When an individual is exposed to misinformation, increased deliberation and controlled thinking to process this information can lead to more accurate detection of fake news. This can occur due to motivated *system 2* reasoning, a part of the dual-process theory, which argues that analytical thinking can override an individual’s intuitive and automatic response to information [22]. Hence, individuals who have high cognitive ability are more likely to deliberate carefully and be skeptical of misinformation [18]. Therefore, we propose the following hypothesis: *H3*, cognitive ability will be negatively associated with (1) perceived accuracy and (2) sharing intentions of COVID-19 misinformation.

We build on these findings and also consider whether personality traits may illustrate further individual differences in misinformation sharing behavior during the COVID-19 pandemic. Some scholars have suggested that personality traits influence misinformation engagement [29]. We refer to the 5-factor model of personality, which encompasses predispositions to everyday experiences and decision-making through the use of lay adjectives [30]. People’s scores of the *big five* personality traits remain relatively stable throughout their lives [31], and scholars have reported replicable relationships of personality traits with different facets of everyday decision-making, including how individuals engage with information, with contrasting findings regarding the role of all personality traits in better news discernment [32,33]. While individual traits do play a role in understanding how individuals engage with information, an overall disposition to manifest “extreme variants” of traits has been linked to compulsive behavior [34], and psychopathy and personality disorders [35]. In the COVID-19 context, personality traits, such as neuroticism, are found to drive beliefs in COVID-19 misinformation and conspiracy theories [36]. We argue that personality traits can offer interesting insights into misinformation engagement in the COVID-19 context, both in terms of themselves and as general indicators of populations with compulsive behavioral tendencies or personality disorders. Furthermore, given the existing literature on the relationship between social media use and personality traits [37,38], there is a need for further research that explores the role of personality traits in COVID-19 misinformation engagement on social media. Hence, we propose the following set of research questions: *RQ1*, “How are personality traits associated with (1) perceived accuracy and (2) sharing intentions of COVID-19 misinformation?” and *RQ2*, “How do personality traits and cognitive ability moderate the mediated relationship between social media news use and sharing intention of COVID-19 misinformation through perceived accuracy?”

To summarize, while the literature on COVID-19 misinformation is growing, there are several gaps that require attention. First, existing studies do not discern between general social media use and news consumption behavior. Social media news consumption behavior is key to understanding misinformation related to COVID-19, as individuals are repeatedly exposed to false information embedded in news stories, particularly from far-right sources [39]. Moreover, social media news use has been linked to COVID-19 vaccine hesitancy, which is a key issue for policy makers worldwide [40]. Second, while rare investigations have focused on the role of personality traits in COVID-19 misinformation engagement on social media [36,41], it has not been studied in conjunction with cognition or cognitive ability, another important factor related to individual engagement with misinformation [42]. Prior research has argued that people with certain personality traits and cognitive ability may engage with misinformation. Specifically, recent studies have found that those with lower cognitive ability are more likely to share false information [17,18]. However, research in this area needs to be expanded upon by scholars. This is also essential because to unravel the psychology of misinformation engagement and to devise counter strategies, we need to consider the individual differences in both personalities and cognition. Lastly, the vast majority of the current literature focuses on Western democracies [8,40,43,44] and largely ignores Asian contexts other than China [45,46]. The Asian population makes up a large portion of social media traffic globally and may be exposed to large amounts of misinformation related to COVID-19, and in turn, this population may share and believe in the false information. For example, according to Statista [47], the estimated number of social media users in Singapore in 2020 was 5.18 million, and this number is expected to increase to 5.68 million by 2025. Moreover, around 83% of Singaporeans seek news online. With such a large number of individuals seeking news online, they may encounter large amounts of false or misleading information. Indeed, approximately 60% of Singaporeans have reported encountering fake news on social media [48]. As such, individuals in Singapore may have been potentially exposed to a large amount of misinformation during COVID-19. This is problematic as Asian countries, such as Singapore, have experienced some of the worst COVID-19 outbreaks. Thus, more attention toward social media misinformation engagement related to COVID-19 is required in Asian contexts.

**Figure 1:**
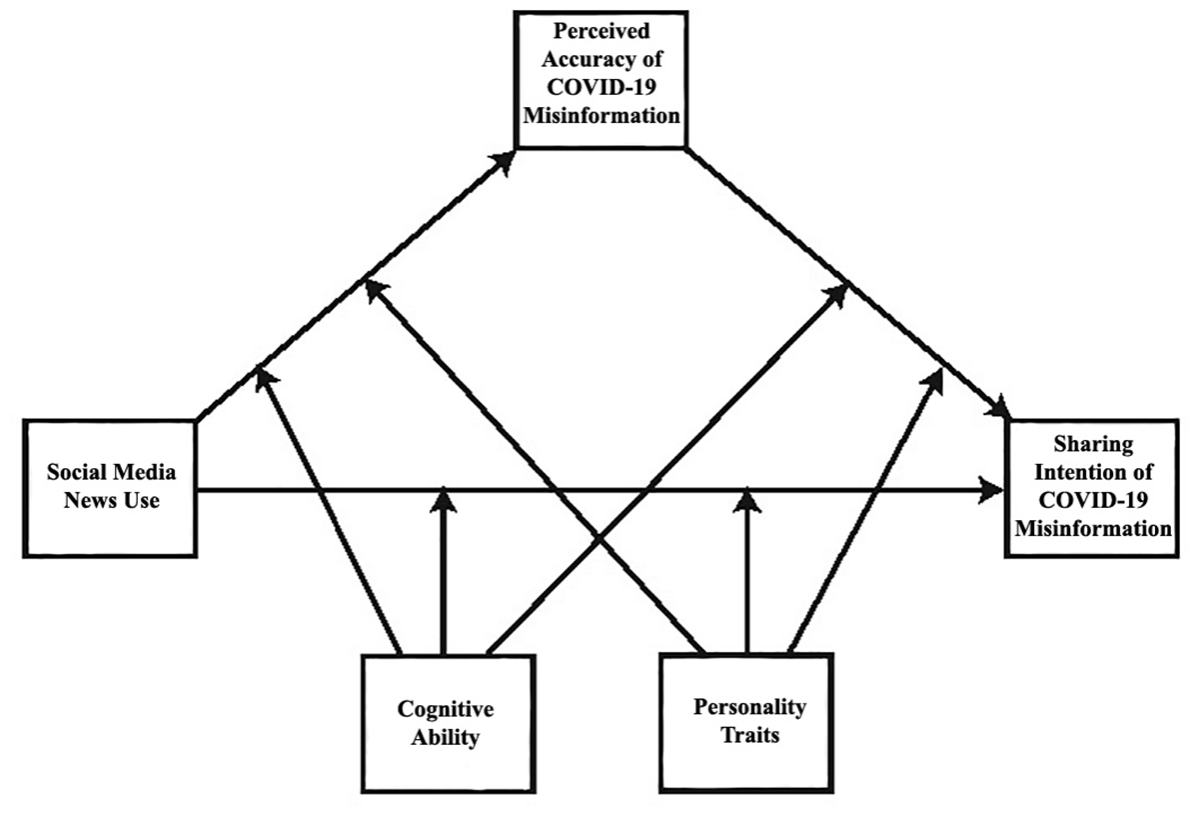
Conceptual framework of the relationship among social media news use, and perceived accuracy and sharing intention of COVID-19 misinformation, with cognitive ability and personality traits as moderators.

In addressing the existing research gaps, this study offers a conceptual framework that explains how social media news use influences COVID-19 misinformation engagement. Precisely, we argue that social media news use leads to COVID-19 misinformation sharing through the induction of belief in misinformation (a mediated relationship). Further, we argue that these effects are dependent on the cognition and personality traits of social media users (Figure 1 illustrates the conceptual framework). By identifying individuals who may be more vulnerable to engagement with COVID-19 misinformation, scientists and policy makers can develop strategies to mitigate the harmful effects of false information and encourage compliance with preventative measures. We focus on cognitive ability and personality traits because they have been associated with misinformation engagement in existing literature [18,32]. Moreover, cognitive ability and personality traits are 2 important psychological mechanisms that can influence behavior. Therefore, both personality traits and cognitive ability can provide important insights into COVID-19 misinformation engagement on social media. However, it is also possible that personality traits and cognitive ability affect each other and jointly impact COVID-19 misinformation engagement. In fact, the findings of a recent study suggest that personality traits and cognitive ability interact and affect political misinformation engagement [49]. Specifically, the study found that low cognitive individuals with certain personality traits, such as neuroticism and openness, were more susceptible to engaging with political misinformation. Accordingly, we extend this body of literature by assessing the impact of personality traits and cognitive ability on misinformation engagement in the context of COVID-19.

In order to extend current research, this study relies on survey data from Singapore for several reasons. First, Singapore is an important country in Asia with a diverse population. Second, although a small country, Singapore is one of the most densely populated countries in Asia and the world [50]. Third, according to a recent report by the Reuters Institute, 53% of Singaporeans relied on social media for news during COVID-19 [51], which may have resulted in increased exposure to misinformation related to COVID-19. Lastly, Singapore’s diverse population bridges many Asian cultures owing to a large number of migrant workers present in the country. We used a quota sampling strategy based on population demographics, which can allow for increased generalizability of findings focused on how social media news may lead to COVID-19 misinformation sharing through the induction of beliefs in misinformation. Moreover, it allows for an examination of how personality traits and cognitive ability moderate the relationships mentioned above. This study ultimately meaningfully contributes to the large body of literature focused on COVID-19 misinformation sharing and belief on social media in an understudied context.

## Methods

### Recruitment

The respondents in the study were recruited through an online panel administered by Qualtrics. Qualtrics is an online survey platform that maintains a panel of several million US residents who have volunteered to take part in online surveys. Respondents who complete a survey are compensated by Qualtrics. Qualtrics uses a quota sampling technique to identify and match participants with the study’s requirement, with the aim to recruit a sample that closely matches the demographic distribution of the census. We used a similar approach to match the sample to population parameters focusing on age and gender. Such techniques have been used previously to ensure that the findings generalize to the larger population [8,17,18,52].

The survey was conducted in March 2022, and out of 1726 respondents who landed on the survey page, 500 respondents answered the survey (28.97% response rate). The study included Singaporean residents older than 21 years. We focused on respondents 21 years or above since this is considered the legal adult age in Singapore.

### Procedure

After providing consent to participate in this study, the participants first answered questions related to their demographic characteristics, media use habits, cognitive ability, and personality traits. Next, the participants proceeded to the (misinformation) evaluation task. They were informed that they would be presented with a few trending news headlines on social media related to COVID-19. Their task involved carefully reading each news headline and answering related questions before moving to the following headline. All 5 viral news headlines presented to the respondents (reported in the Measures section) were false, according to factchecking websites. The participants were not informed that the headlines were not true, as this would have affected the study findings [53].

### Ethics Approval

The Institutional Review Board at Nanyang Technological University approved the study protocol (IRB-2022-097).

### Measures

Perceived accuracy of COVID-19 misinformation was measured by asking the respondents to rate their level of perceived accuracy (1 [not at all accurate] to 5 [extremely accurate]) for the 5 claims in the news headlines. The scale is based on previous research on the perceived accuracy of news/misinformation headlines [54,55]. The participants were asked how accurate are the claims that (1) coconut is effective in reducing COVID-19 symptoms; (2) the pH miracle lifestyle healing program of alkaline diet, exercise, and healing foods can cure COVID-19; (3) COVID vaccines are dangerous and ineffective against the Omicron variant; (4) mRNA COVID-19 vaccinations cause magnetism by introducing graphene oxide into the blood; and (5) there is no evidence of the COVID-19 virus and no one has isolated and sequenced SARS-CoV-2 from any patient sample. The responses to the 5 items were averaged to create an index of the perceived claim accuracy of misinformation regarding COVID-19 (mean 2.01, SD 1.03; α=.91).

Sharing intention of COVID-19 misinformation was measured by asking respondents how likely (1 [extremely likely to share] to 5 [not at all likely to share]) are they to share these news headlines on their social media profiles. While it is acknowledged that these sharing intentions are hypothetical, such approaches have been previously adopted by scholars to measure misinformation sharing [54,56]. Moreover, self-reports of sharing intentions have been found to be strongly associated with attention received by news headlines on social media [57]. The responses to sharing intentions were reverse coded, so a higher value represents greater sharing intention. The responses were then averaged to create an index of the sharing intention of COVID-19 misinformation (mean 1.96, SD 1.08; α=.93).

Social media news use was measured by asking respondents how frequently (1 [never] to 5 [daily]) do they engage in the following: (1) post on their timeline about political or public affairs news; (2) share posts about political or public affairs news; (3) comment on posts about political or public affairs news; (4) read their news feed about political or public affairs news; and (5) read the news feed/timelines of friends about political or public affairs news [58]. The responses to the 5 items were averaged to create an index of social media news use (mean 2.26, SD 0.90; α=.79).

Cognitive ability was measured by the wordsum test. The test includes 10 questions, where participants are provided with a source word (eg, caprice) and their task involves matching the source word with the closest related word from a list of 5 target words; in this case, the 5 words are (1) value, (2) star, (3) grimace, (4) whim, and (5) inducement. The correct responses to the 10 questions were summed to create a scale of cognitive ability (mean 5.48, SD 2.48; α=.76). While the test is vocabulary based, it has high covariance with general intelligence [59,60] and has been frequently used by scholars to investigate the role of cognitive ability in misinformation engagement [17,18,61,62]. The test is also applicable in Singapore since English is the primary language of the educational system and is also the most commonly used verbal language in the country [63].

Personality traits were measured through a total of 10 statements asking respondents to rate their level of agreement (1 [strongly disagree] to 7 [strongly agree]) for the given statements (eg, “I see myself as someone who worries a lot,” “I see myself as someone who is talkative,” and “I see myself as someone who does a thorough job”). The responses were combined to cover 5 different personality traits, including neuroticism (mean 4.41, SD 1.54; α=.88), extraversion (mean 4.11, SD 1.45; α=.80), openness (mean 4.81, SD 1.17; α=.77), agreeableness (mean 5.06, SD 1.08; α=.72), and conscientiousness (mean 5.24, SD 1.06; α=.77).

### Statistical Analysis

We employed ordinary least squares (OLS) regression models to test the effect of social media news use on perceived accuracy and sharing intentions of COVID-19 misinformation. We ran a mediation model to explore the proposed mediation relationship. Finally, we employed a conditional moderated mediation analysis (using the SPSS PROCESS macro v 3.5) [64] to examine the moderating role of cognitive ability and personality traits in the mediation process.

We also controlled for several variables, including demographics, traditional media news use (television, radio, and print news use averaged; 1 [never] to 5 [daily]; mean 2.83, SD 1.20; α=.73), and political interest (1 [not at all interested] to 5 [extremely interested]; mean 2.82, SD 1.06). Traditional media news use and political interest were included as covariates since they have been found to be important factors in sharing misinformation [17,18,29].

Demographics included (1) age (mean 39.23, SD 14.12 years), (2) gender (51% female), (3) education (1 [no formal education] to 7 [doctoral degree]; mean 4.47, SD 0.99; median Bachelor’s degree), (4) household income (1 [less than SGD $1000] to 11 [more than SGD $20,000]; mean 5.07, SD 2.60; median SGD $7000-$8999), and (5) race (76.6% Chinese majority). A currency exchange rate of SGD $1=US $710.98 is applicable.

## Results

**Figure 2:**
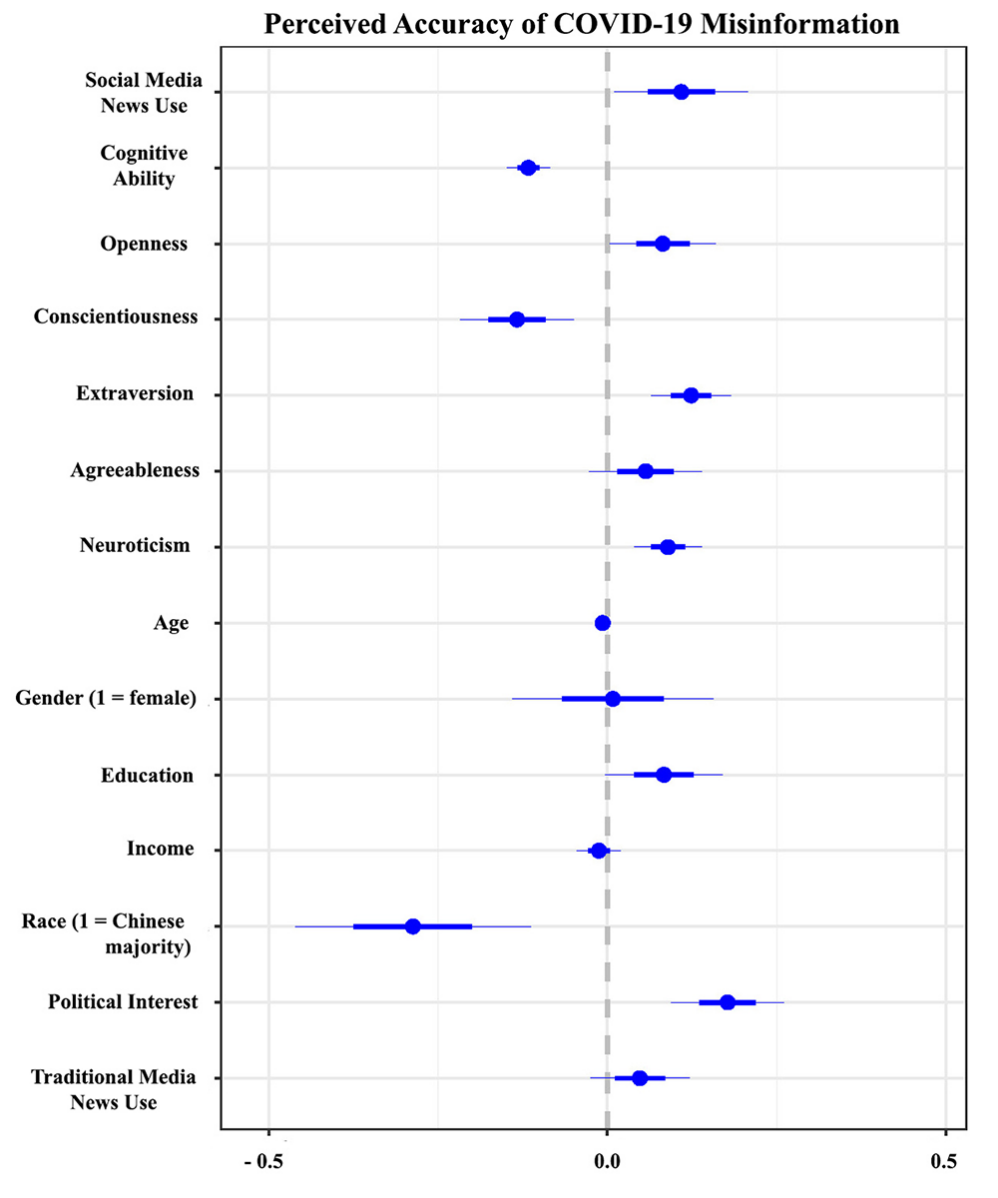
Predicting perceived accuracy of COVID-19 misinformation. The plot includes regression coefficients for all variables.

**Figure 3:**
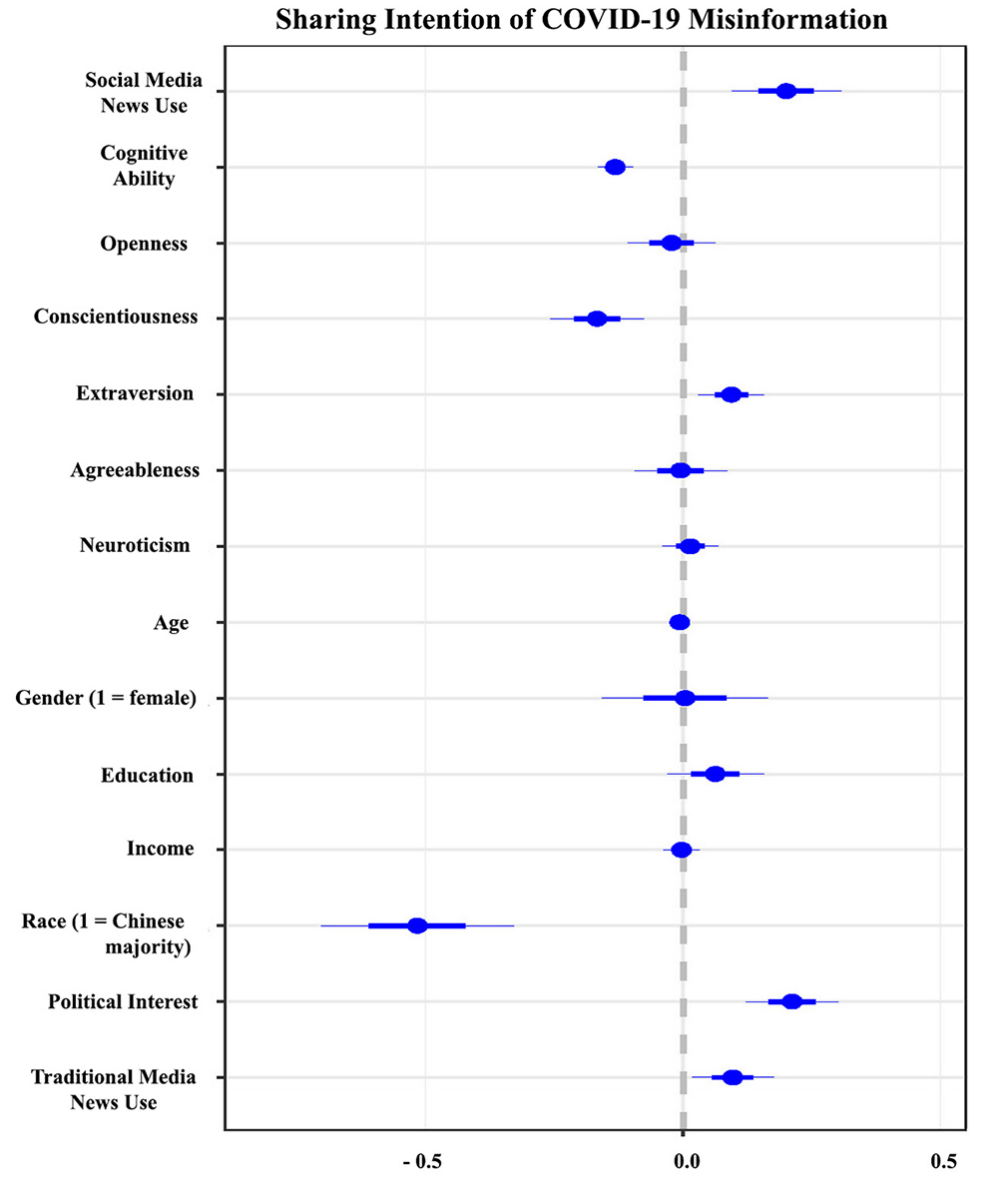
Predicting sharing intention of COVID-19 misinformation. The plot includes regression coefficients for all variables.

In the first step, we ran regression analyses to predict perceived accuracy and sharing intentions. The results of the OLS regression are plotted in Figures 2 and 3 (see Multimedia Appendix 1 for more details). The results suggested that those who were younger (perceived accuracy: B=−0.007, SE=0.003; *P*=.03; sharing intention: B=−0.006, SE=0.003; *P*=.05) and had higher political interest (perceived accuracy: B=0.177, SE=0.042; *P*<.001; sharing intention: B=0.212, SE=0.045; *P*<.001) were likely to both perceive the misinformation to be accurate and share it on social media.

Among the variables of interest, we observed that those who frequently relied on social media for news consumption were likely to not only perceive the misinformation to be accurate (B=0.109, SE=0.049; *P*=.03) but also share it on social media (B=0.200, SE=0.053; *P*<.001). We also observed that those with high cognitive ability were less likely to perceive the false claims to be true (B=−0.117, SE=0.016; *P*<.001) and share them (B=−0.132, SE=0.017; *P*<.001).

The personality correlates suggested that extraverted individuals were likely to not only perceive the misinformation to be accurate (B=0.124, SE=0.029; *P*<.001) but also show higher sharing intentions (B=0.094, SE=0.032; *P*<.001). Conversely, we observed that conscientious individuals were less likely to perceive the misinformation to be accurate (B=−0.133, SE=0.042; *P*<.001) or share it (B=−0.167, SE=0.045; *P*<.001). In addition, it was also observed that neurotic (B=0.089, SE=0.025; *P*<.001) and open individuals (B=0.082, SE=0.039; *P*=.04) were more likely to perceive the claims to be accurate.

Next, to explore the mechanism of how social media news use induces misinformation sharing intentions through perceived accuracy, we ran mediation analyses using the SPSS PROCESS macro [64], with social media news use as the predictor variable, perceived accuracy as the mediator, and sharing intention as the outcome variable. The bootstrapping method was used to estimate the indirect effects (N=5000).

**Figure 4:**
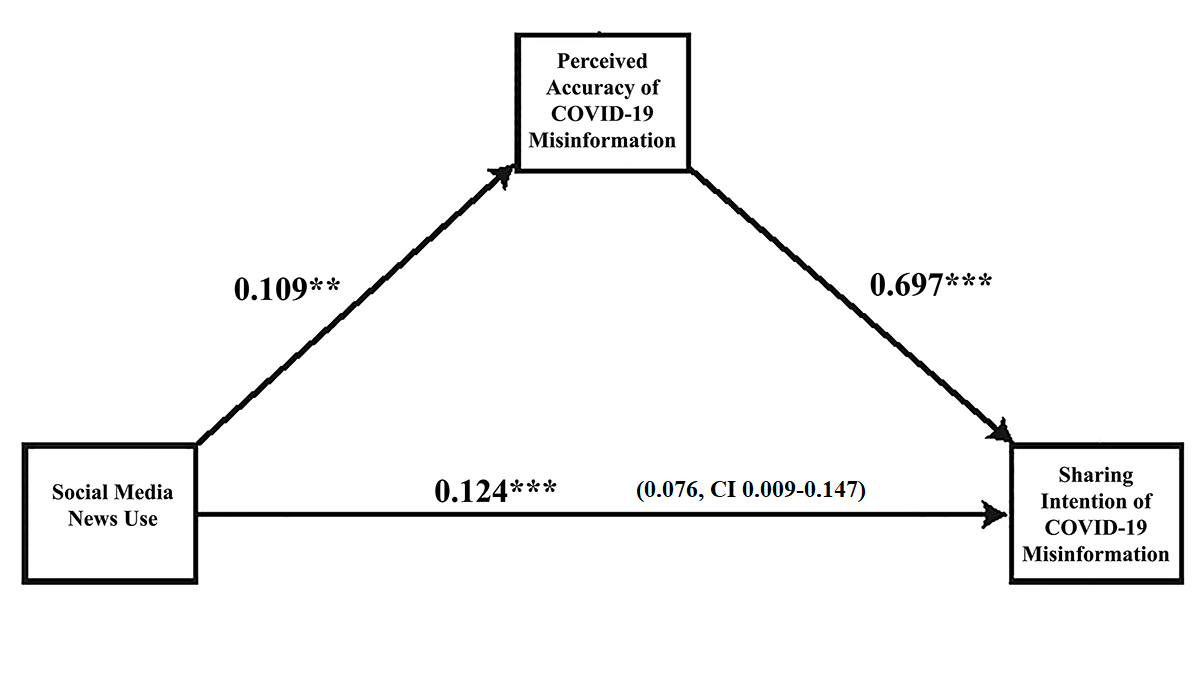
Illustrated mediation of social media news use, perceived accuracy, and sharing intention of COVID-19 misinformation. Estimates are calculated using the SPSS PROCESS macro (Model 4) [64]. The number in parenthesis is the indirect effect with lower limit CI to upper limit CI. Bootstrap resample=5000. Statistical controls include age, gender, education, income, race, political interest, traditional media news use, personality traits, and cognitive ability. **P<.01, ***P<.001.

The results are illustrated in Figure 4. As observed, we found that social media news use (B=0.109, SE=0.049, 95% CI 0.012-0.206) was positively associated with the perceived accuracy of misinformation, which concurrently was positively associated with sharing intentions of misinformation (B=0.697, SE=0.038, 95% CI 0.623-0.771). The direct relationship between social media news use and sharing intentions was also found to be significantly positive (B=0.124, SE=0.041, 95% CI 0.044-0.205).

A formal statistical test of the mediation process suggested that the indirect effects were statistically significant (B=0.076, SE=0.036, 95% CI 0.009-0.147). These results indicated that social media news use translates into sharing intentions of misinformation as individuals perceive this misinformation to be accurate.

Finally, we explored how cognitive ability and personality traits moderated the relationship between social media news use and misinformation sharing intention through perceived accuracy of misinformation. We employed conditional process analyses using the SPSS PROCESS macro for 2 conditional moderators (model 76) [64]. The results of the conditional indirect effects of social media news use on sharing intentions via perceived claim accuracy at different levels (−1 SD, mean, and +1 SD) of cognitive ability and individual personality traits are included in Table 1. While exploring the effects of each personality trait (eg, openness), we used the other 4 components (eg, conscientiousness, extraversion, agreeableness, and neuroticism) as controls in the specific models (see Multimedia Appendix 2 for more details).

The patterns across the 5 personality traits at high levels of cognitive ability suggested that none of the indirect effects (except high cognitive ability and high extraversion) were statistically significant. The general implication is that personality traits do not significantly influence misinformation engagement at higher levels of cognitive ability. On the contrary, it was also found that at lower levels of cognitive ability, individuals who displayed heightened levels of *any* personality trait were more likely to engage with misinformation. In general, this suggests that individuals with low cognitive ability are more susceptible to misinformation if they appear to demonstrate a compulsive outlook toward any of the personality traits.

**Table 1:** Conditional indirect effects of social media news use on sharing intentions through perceived accuracy at different levels of cognitive ability and personality traits.

Cognitive ability level and personality trait level^a^	Effect	Boot SE^b^	LLCI^c^		ULCI^d^
Low (−1 SD) cognitive ability and low (−1 SD) openness	0.12	0.07	−0.01		0.25
Low (−1 SD) cognitive ability and mean openness	0.15^e^	0.04	0.07		0.24
Low (−1 SD) cognitive ability and high (+1 SD) openness	0.18^e^	0.05	0.10		0.28
Mean cognitive ability and low (−1 SD) openness	0.03	0.06	−0.09		0.14
Mean cognitive ability and mean openness	0.07	0.04	−0.01		0.15
Mean cognitive ability and high (+1 SD) openness	0.11^e^	0.05	0.03		0.21
High (+1 SD) cognitive ability and low (−1 SD) openness	−0.08	0.07	−0.22		0.05
High (+1 SD) cognitive ability and mean openness	−0.03	0.05	−0.14		0.07
High (+1 SD) cognitive ability and high (+1 SD) openness	0.01	0.06	−0.10		0.14
Low (−1 SD) cognitive ability and low (−1 SD) conscientiousness	0.07	0.05	−0.01	0.19	
Low (−1 SD) cognitive ability and mean conscientiousness	0.14^e^	0.04	0.07	0.23	
Low (−1 SD) cognitive ability and high (+1 SD) conscientiousness	0.24^e^	0.05	0.13	0.35	
Mean cognitive ability and low (−1 SD) conscientiousness	−0.01	0.05	−0.09	0.10	
Mean cognitive ability and mean conscientiousness	0.06	0.04	−0.01	0.14	
Mean cognitive ability and high (+1 SD) conscientiousness	0.15^e^	0.05	0.05	0.26	
High (+1 SD) cognitive ability and low (−1 SD) conscientiousness	−0.11	0.06	−0.22	0.00	
High (+1 SD) cognitive ability and mean conscientiousness	−0.05	0.05	−0.15	0.04	
High (+1 SD) cognitive ability and high (+1 SD) conscientiousness	0.03	0.07	−0.11	0.16	
Low (−1 SD) cognitive ability and low (−1 SD) extraversion	−0.01	0.04	−0.09	0.08	
Low (−1 SD) cognitive ability and mean extraversion	0.09^e^	0.04	0.02	0.18	
Low (−1 SD) cognitive ability and high (+1 SD) extraversion	0.23^e^	0.05	0.15	0.33	
Mean cognitive ability and low (−1 SD) extraversion	−0.06	0.04	−0.13	0.01	
Mean cognitive ability and mean extraversion	0.05	0.03	−0.02	0.12	
Mean cognitive ability and high (+1 SD) extraversion	0.21^e^	0.05	0.11	0.30	
High (+1 SD) cognitive ability and low (−1 SD) extraversion	−0.13^e^	0.05	−0.23	−0.05	
High (+1 SD) cognitive ability and mean extraversion	−0.01	0.05	−0.11	0.09	
High (+1 SD) cognitive ability and high (+1 SD) extraversion	0.16^e^	0.08	0.01	0.31	
Low (−1 SD) cognitive ability and low (−1 SD) agreeableness	0.08	0.05	−0.02	0.19	
Low (−1 SD) cognitive ability and mean agreeableness	0.14^e^	0.04	0.07	0.23	
Low (−1 SD) cognitive ability and high (+1 SD) agreeableness	0.21^e^	0.05	0.12	0.31	
Mean cognitive ability and low (−1 SD) agreeableness	−0.01	0.04	−0.09	0.09	
Mean cognitive ability and mean agreeableness	0.07^e^	0.04	0.00	0.14	
Mean cognitive ability and high (+1 SD) agreeableness	0.14^e^	0.05	0.04	0.25	
High (+1 SD) cognitive ability and low (−1 SD) agreeableness	−0.11	0.05	−0.22	0.00	
High (+1 SD) cognitive ability and mean agreeableness	−0.03	0.05	−0.13	0.07	
High (+1 SD) cognitive ability and high (+1 SD) agreeableness	0.05	0.07	−0.08	0.20	
Low (−1 SD) cognitive ability and low (−1 SD) neuroticism	0.11^e^	0.06	0.01	0.23	
Low (−1 SD) cognitive ability and mean neuroticism	0.15^e^	0.04	0.07	0.23	
Low (−1 SD) cognitive ability and high (+1 SD) neuroticism	0.19^e^	0.05	0.10	0.29	
Mean cognitive ability and low (−1 SD) neuroticism	0.04	0.04	−0.04	0.12	
Mean cognitive ability and mean neuroticism	0.08^e^	0.04	0.01	0.15	
Mean cognitive ability and high (+1 SD) neuroticism	0.12^e^	0.06	0.02	0.25	
High (+1 SD) cognitive ability and low (−1 SD) neuroticism	−0.06	0.05	−0.16	0.03	
High (+1 SD) cognitive ability and mean neuroticism	−0.01	0.06	−0.12	0.10	
High (+1 SD) cognitive ability and high (+1 SD) neuroticism	0.04	0.08	−0.11	0.21	

^a^Analyses were performed using the PROCESS macro for SPSS (model 76), applying 5000 bootstrap samples. Statistical controls include age, gender, education, income, race, political trust, political interest, traditional media news use, and personality traits as well as the remaining 4 personality traits.

^b^SE: standard error.

^c^LLCI: lower limit CI.

^d^ULCI: upper limit CI.

^e^Statistically significant effect.

## Discussion

### Principal Findings

Numerous studies have explored public engagement with COVID-19 misinformation on social media [7,11,13,14,36], but not many have explored how news consumption through social media platforms affects such misinformation engagement. This study aimed to explore the mechanism of how social media news use influences believing and sharing COVID-19 misinformation. It also examined individual differences in such engagement through the lens of cognitive and personality factors.

The results of this study are critical in the context of the COVID-19 information environment on social media. A wide majority of the population across societies, including Singapore, rely on social media as a critical source of news and information. More recently, the pandemic made social media platforms relevant as sources of COVID-19 information. Against this background, an association between news consumption via social media and misinformation engagement raises concerns regarding the utility of these platforms as information sources.

The results suggest that frequent reliance on social media for news consumption is associated with increased belief and sharing intentions of COVID-19 misinformation. Moreover, the mediation results indicate that social media news users who believe the COVID-19 misinformation to be accurate are more likely to share it. Furthermore, the study found that personality traits do not significantly influence misinformation sharing at higher levels of cognitive ability. Therefore, high cognitive individuals are less likely to believe or share misinformation irrespective of personality traits. On the contrary, at lower levels of cognitive ability, those with high levels of all personality traits are more vulnerable to COVID-19 misinformation sharing.

The plethora of news and information on social media creates a system of information overload. Given that most users have cognitive biases and do not engage in critical information processing, it is likely that such information overload could explain why increased social media news use is associated with the belief and sharing of COVID-19 misinformation. Indeed, scholars have argued that people fail to think sufficiently before engaging with COVID-19 misinformation [65].

The findings highlight the risks associated with news consumption via social media platforms, but we also found that the observed associations vary by the personality and cognitive ability of individuals. We observed that some personality traits (eg, extraversion and conscientiousness) were associated with sharing intentions but others were not (eg, neuroticism, openness, and agreeableness). However, further probing suggested that the effects of personality traits on sharing intents are driven mainly by low rather than high cognitive social media news users. These results are in line with recent findings where cognitive ability was found to be positively associated with better truth discernment [54,55], weaker belief in false content [17,18,66], and reduced sharing intention of misinformation [56]. In addition, a higher cognitive ability allows individuals to make better risk assessments and filter what information is relevant when placing their trust [67]. Thus, it seems that high cognitive individuals, largely irrespective of their personality traits, are better at processing the false information presented to them and thereby refrain from sharing.

It is important to consider how together with the lack of cognitive ability, a display of high-valued personality traits can further amplify the vulnerability of individuals toward misinformation sharing and engagement. Individuals with low cognitive ability, yet with an extreme tendency to be open to experience, conscientious, extraverted, agreeable, or neurotic, are more likely to fall for and share misinformation. The findings corroborate previous work [33], which has reported on the association of all personality traits with the tendency to believe misinformation; however, we illustrate the importance of cognitive ability as a differentiator in this relationship.

It is important to note here that this relationship is not causal and there are other possible causal relationships that may impact COVID-19 misinformation engagement. For instance, a recent study found evidence of relationships between incidental news exposure on social media, news literacy, and COVID-19 misperceptions [68]. The presence or absence of news literacy could impact how people process news about COVID-19, which, in turn, could dictate how they engage with news related to COVID-19 [69]. Other studies have also found scientific knowledge to play a role in COVID-19 misinformation engagement [65]. Since it has become a politicized issue, ideology could also impact how people engage with COVID-19 misinformation. In fact, political conservatism has been found to be associated with belief in COVID-19 misinformation [15]. Moreover, some social media users are exposed to more misinformation than others, which can lead them to be more susceptible to misinformation through the illusory truth effect. Thus, it could be that individuals who are more susceptible to misinformation also engage with COVID-19 misinformation differently. The unabated spread of misinformation during a destructive global pandemic, such as COVID-19, has raised complex issues and problems. As such, there are a variety of possible factors that can lead to COVID-19 misinformation engagement. Thus, more work is needed to establish causal links with COVID-19 misinformation engagement.

Moreover, the findings presented here are specific to the context of COVID-19 misinformation. While the results on social media news use and cognitive ability are largely consistent with previous literature [17,18], it is to be explored if the patterns for personality traits and the interaction with cognitive ability are consistent across other forms of misinformation.

### Practical Implications

This study has practical implications for those who rely on social media for news. Existing studies have found evidence of widespread misinformation related to COVID-19 on different social media platforms, such as YouTube, Twitter, Instagram, and Reddit [6]. Our results add an additional layer of concern for social media platforms when it comes to combatting COVID-19 misinformation. Some studies have found accuracy nudges to be successful in correcting COVID-19 misinformation as a result of news exposure [65]. However, more work is required in this area to fully understand and develop strategies to fight COVID-19 misinformation and its pernicious effects in contexts other than the United States. While our findings represent active social media news users, they also have implications for those who do not primarily use social media for news consumption. For example, a recent survey found that most online users are exposed to news about COVID-19 on social media even when they are on social media for different purposes [65]. Such incidental exposure to COVID-19 information could also expose these individuals to misinformation, thereby furthering the adverse consequences. In fact, a recent study found that incidental news exposure was related to COVID-19 misperceptions [68].

Additionally, previous studies have found that the older population is more vulnerable to believing and sharing misinformation [20,70]. However, we found that younger respondents were more likely to believe and share COVID-19 misinformation. These results are in line with studies that have found similar patterns [71,72]. For example, a study involving samples from 5 countries found that older individuals were less susceptible to misinformation about COVID-19 [15]. Younger respondents may likely lack the necessary skills and abilities to discern COVID-19 misinformation despite high literacy rates, since it is specialized health information. Further, our results have implications for individuals with lower levels of cognitive ability. For example, a study found that lower cognitive ability is related to increased susceptibility to deepfakes on social media. Furthermore, scholars have argued that people often fail to think sufficiently about the accuracy of content on social media before they share it [22]. As such, those with low cognitive ability could be more at risk to not only believe in COVID-19 misinformation, but also share it with their networks on social media platforms. Thus, researchers and social media platforms must find ways to counter the widespread misinformation related to COVID-19 to promote compliance with public health protocols.

### Limitations

The results are based on cross-sectional data and limit any causal inferences. While the findings confirm the overall consensus of the impact of social media on misinformation engagement during COVID-19, future scholars should collect longitudinal data to make causal arguments. The findings are based on a single context where social media penetration is high and strict governmental regulations largely control misinformation. Therefore, how these findings would apply to societies with low social media penetration remains unanswered. Next, while our operationalization of social media news use (focusing on political and public affairs news) is consistent with a majority of the literature [58,73], it remains to be seen how the effects of social media news would differ based on other forms of news use (eg, health news). Finally, we focused on one aspect of cognitive ability (through the lens of verbal reasoning). Others may consider comparing the effects of different types of intelligence (eg, fluid vs crystallized) since various forms of cognitive ability may have differential impacts.

### Conclusion

Social media platforms are increasingly being used as news aggregators and primary news sources by citizens worldwide. Individual differences in user behavior can lead to users being less or more vulnerable to misinformation engagement, and individuals with low cognitive ability and compulsive personality traits are at a further disadvantage as compared with others. We recommend that policymakers and social media giants should consider targeted interventions that aim at understanding and checking patterns in everyday behavior that could amplify individual risk of encountering or sharing misinformation. We also recommend experiments with interventions to curb the spread of COVID-19 misinformation.

## Appendix

Multimedia Appendix 1 Predicting the perceived accuracy and sharing intention of COVID-19 misinformation.

Multimedia Appendix 2 Conditional direct effects of social media news use on sharing intentions through perceived accuracy at different levels of cognitive ability and personality traits.

## Abbreviations

OLS: ordinary least squares
