# Supplementary material for: Social Media News Use and COVID-19 Misinformation Engagement: Survey Study
Source: J Med Internet Res. 2022 Sep 20;24(9):e38944. doi: 10.2196/38944 (PMC9533200; doi:10.2196/38944)
Supplement: Multimedia Appendix 1 [file jmir_v24i9e38944_app1.docx]

**Multimedia Appendix 1.** Predicting the perceived accuracy and sharing intention of COVID-19 misinformation.^a^

| Predictor Variables | Perceived Accuracy | | Sharing Intention | |
| --- | --- | --- | --- | --- |
|  | B | SE | B | SE |
| **Controls** |  |  |  |  |
| Age | -.007* | .003* | -.006* | .003* |
| Gender | .008 | .074 | .004 | .003 |
| Education | .083 | .043 | .063 | .047 |
| Income | -.013 | .016 | -.003 | .017 |
| Race | -.287*** | .087*** | -.516*** | .094*** |
| Political interest | .177*** | .042*** | .212*** | .045*** |
| Traditional media news use | .048 | .037 | .097* | .039* |
| **Independent Variables** |  |  |  |  |
| Social media news use | .109* | .049* | .200*** | .053*** |
| Openness | .082* | .039* | -.022 | .042 |
| Conscientiousness | -.133** | .042** | -.167*** | .045*** |
| Extraversion | .124*** | .029*** | .094*** | .032*** |
| Agreeableness | .056 | .042 | -.005 | .045 |
| Neuroticism | .089*** | .025*** | .014 | .027 |
| Cognitive Ability | -.117*** | .016*** | -.132*** | .017*** |
| **Total R^2^** | 45.4 |  | 42.6 |  |

^a^ Statistical significance is marked as **p* < 0.05; ***p* < 0.01; ****p* < 0.001.
