# Supplementary material for: Social Media News Use and COVID-19 Misinformation Engagement: Survey Study
Source: J Med Internet Res. 2022 Sep 20;24(9):e38944. doi: 10.2196/38944 (PMC9533200; doi:10.2196/38944)
Supplement: Multimedia Appendix 2 [file jmir_v24i9e38944_app2.docx]

**Multimedia Appendix 2.** Conditional direct effects of social media news use on sharing intentions through perceived accuracy at different levels of cognitive ability and personality traits ^a-c^

| Cognitive Ability | Openness | Effect | Boot SE | LLCI | | ULCI | |
| --- | --- | --- | --- | --- | --- | --- | --- |
| Low (-1 SD) | Low (-1 SD) | 0.21^***^ | 0.07 | 0.06 | | 0.35 | |
|  | Mean | 0.21^***^ | 0.06 | 0.10 | | 0.32 | |
|  | High (+1 SD) | 0.21^***^ | 0.06 | 0.09 | | 0.32 | |
| Mean | Low (-1 SD) | 0.13^***^ | 0.06 | 0.01 | | 0.25 | |
|  | Mean | 0.13^***^ | 0.04 | 0.04 | | 0.21 | |
|  | High (+1 SD) | 0.12^***^ | 0.05 | 0.03 | | 0.22 | |
| High (+1 SD) | Low (-1 SD) | 0.04 | 0.07 | -0.09 | | 0.18 | |
|  | Mean | 0.04 | 0.05 | -0.06 | | 0.15 | |
|  | High (+1 SD) | 0.04 | 0.06 | -0.08 | | 0.16 | |
|  |  |  |  |  | |  | |
| Cognitive Ability | Conscientiousness | Effect | Boot SE | LLCI | ULCI | |  |
| Low (-1 SD) | Low (-1 SD) | 0.26^***^ | 0.07 | 0.12 | 0.39 | |  |
|  | Mean | 0.21^***^ | 0.05 | 0.10 | 0.32 | |  |
|  | High (+1 SD) | 0.17^***^ | 0.06 | 0.05 | 0.28 | |  |
| Mean | Low (-1 SD) | 0.17^***^ | 0.06 | 0.06 | 0.29 | |  |
|  | Mean | 0.13^***^ | 0.04 | 0.05 | 0.21 | |  |
|  | High (+1 SD) | 0.08 | 0.05 | -0.01 | 0.18 | |  |
| High (+1 SD) | Low (-1 SD) | 0.09 | 0.07 | -0.04 | 0.23 | |  |
|  | Mean | 0.05 | 0.05 | -0.06 | 0.15 | |  |
|  | High (+1 SD) | 0.00 | 0.06 | -0.11 | 0.12 | |  |
|  |  |  |  |  |  | |  |
| Cognitive Ability | Extraversion | Effect | Boot SE | LLCI | ULCI | |  |
| Low (-1 SD) | Low (-1 SD) | 0.34^***^ | 0.08 | 0.19 | 0.49 | |  |
|  | Mean | 0.25^***^ | 0.06 | 0.14 | 0.36 | |  |
|  | High (+1 SD) | 0.16^***^ | 0.06 | 0.05 | 0.28 | |  |
| Mean | Low (-1 SD) | 0.23^***^ | 0.06 | 0.11 | 0.34 | |  |
|  | Mean | 0.14^***^ | 0.04 | 0.06 | 0.22 | |  |
|  | High (+1 SD) | 0.05 | 0.05 | -0.05 | 0.15 | |  |
| High (+1 SD) | Low (-1 SD) | 0.11 | 0.06 | -0.01 | 0.23 | |  |
|  | Mean | 0.02 | 0.05 | -0.08 | 0.13 | |  |
|  | High (+1 SD) | -0.07 | 0.07 | -0.20 | 0.07 | |  |
|  |  |  |  |  |  | |  |
| Cognitive Ability | Agreeable | Effect | Boot SE | LLCI | ULCI | |  |
| Low (-1 SD) | Low (-1 SD) | 0.16^***^ | 0.07 | 0.02 | 0.30 | |  |
|  | Mean | 0.20^***^ | 0.06 | 0.09 | 0.31 | |  |
|  | High (+1 SD) | 0.24^***^ | 0.06 | 0.12 | 0.35 | |  |
| Mean | Low (-1 SD) | 0.08 | 0.06 | -0.03 | 0.20 | |  |
|  | Mean | 0.12^***^ | 0.04 | 0.04 | 0.20 | |  |
|  | High (+1 SD) | 0.16^***^ | 0.05 | 0.06 | 0.26 | |  |
| High (+1 SD) | Low (-1 SD) | 0.01 | 0.07 | -0.12 | 0.14 | |  |
|  | Mean | 0.04 | 0.05 | -0.06 | 0.15 | |  |
|  | High (+1 SD) | 0.08 | 0.06 | -0.04 | 0.20 | |  |
|  |  |  |  |  |  | |  |
| Cognitive Ability | Neuroticism | Effect | Boot SE | LLCI | ULCI | |  |
| Low (-1 SD) | Low (-1 SD) | 0.13 | 0.08 | -0.02 | 0.28 | |  |
|  | Mean | 0.19^***^ | 0.06 | 0.07 | 0.30 | |  |
|  | High (+1 SD) | 0.24^***^ | 0.06 | 0.12 | 0.36 | |  |
| Mean | Low (-1 SD) | 0.07 | 0.06 | -0.04 | 0.18 | |  |
|  | Mean | 0.13^***^ | 0.04 | 0.05 | 0.21 | |  |
|  | High (+1 SD) | 0.18^***^ | 0.05 | 0.07 | 0.29 | |  |
| High (+1 SD) | Low (-1 SD) | 0.01 | 0.06 | -0.10 | 0.13 | |  |
|  | Mean | 0.07 | 0.06 | -0.04 | 0.18 | |  |
|  | High (+1 SD) | 0.12 | 0.07 | -0.02 | 0.27 | |  |

^a-c.^ Analyses were performed using the PROCESS macro for SPSS (Model 76), applying 5,000 bootstrap samples. Statistical controls include age, gender, education, income, race, political trust, political interest, traditional media news use, and personality traits and other four personality traits. ^***^ statistically significant effects.
